# Two-photon responsive porphyrinic metal-organic framework involving Fenton-like reaction for enhanced photodynamic and sonodynamic therapy

**DOI:** 10.1186/s12951-022-01436-3

**Published:** 2022-05-06

**Authors:** Wenyao Duan, Bo Li, Wen Zhang, Jiaqi Li, Xin Yao, Yupeng Tian, Jun Zheng, Dandan Li

**Affiliations:** 1 Institutes of Physics Science and Information Technology, Key Laboratory of Structure and Functional Regulation of Hybrid Materials, Ministry of Education, Anhui University, Hefei, 230601 People’s Republic of China; 2 Department of Chemistry, Key Laboratory of Functional Inorganic Material Chemistry of Anhui Province, Anhui University, Hefei, 230601 People’s Republic of China

**Keywords:** Metal-organic framework, Two-photon, Hypoxia, Sonodynamic therapy, Photodynamic therapy

## Abstract

Designing new oxygenation nanomaterials by oxygen-generating or oxygen-carrying strategies in hypoxia-associated anti-tumor therapy is a high priority target yet challenge. In this work, we fabricated a nanoplatform involving Fenton-like reaction, Pd@MOF-525@HA, to relieve tumor hypoxia via oxygen-generating strategy for enhanced oxygen-dependent anti-tumor therapy. Thereinto, the porphyrinic MOF-525 can produce singlet oxygen (^1^O_2_) via light or ultrasonic irradiation for photodynamic and sonodynamic therapy. Notably, the well-dispersed Pd nanocubes within MOF-525 can convert H_2_O_2_ into O_2_ to mitigate the hypoxic environment for enhanced therapy outcome. Moreover, the two-photon activity and cancer cell specific targeting capability of Pd@MOF-525@HA gave rise to deeper tissue penetration and near-infrared light-induced fluorescence imaging to achieve precise guidance for cancer therapy. This work provides a feasible way in designing new oxygenation nanomaterials to relieve tumor hypoxia for enhanced cancer treatment.

**Graphical Abstract:**

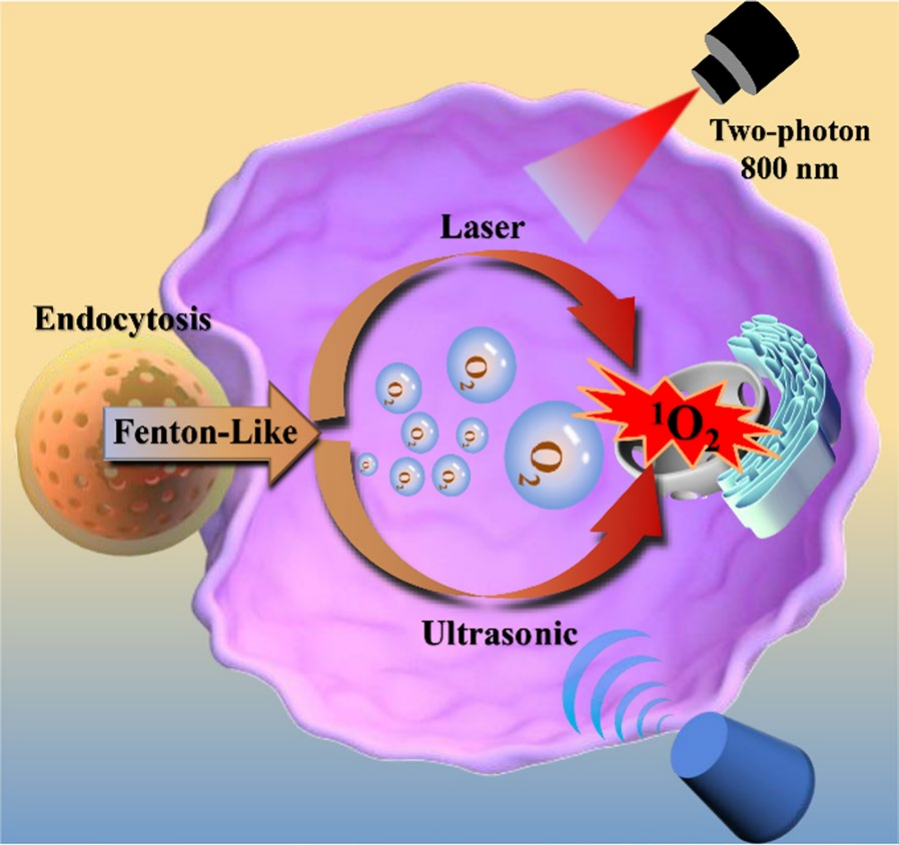

**Supplementary Information:**

The online version contains supplementary material available at 10.1186/s12951-022-01436-3.

## Introduction

As the second leading cause of human death, cancer poses a grievous threat to human health [1, 2]. Facing the characteristics of infinite proliferation and easy metastasis of tumor cells, traditional radiotherapy and chemotherapy in combating cancer cells show unsatisfactory treatment results due to the multidrug resistance and severe side effects [3–5]. In this sense, researchers have made great efforts to develop more effective treatment methods, such as sonodynamic therapy (SDT) and photodynamic therapy (PDT), which are noninvasive tools with high selectivity for local cancers given the generated reactive oxygen species (ROS) from the photo/sonosensitizers by laser/ultrasound irradiation in an aerobic environment [6–12]. Unfortunately, the hypoxic environment of the tumor significantly limits their efficiencies [13–16]. To this end, it is imperative to develop a valid PDT/SDT system that can relieve tumor hypoxia.

Presently, the PDT agents used in clinic are mainly porphyrin derivatives, the excitation wavelength of which are located in the visible region (400–700 nm) leads to poor penetration limiting their further application [17, 18]. However, it is worth mentioning that these porphyrin derivatives can be employed as sonosensitizers to effectively compensate for the barrier of shallow penetration depth in PDT [19, 20]. Notably, the above two cancer treatment methods are highly oxygen dependent, the severe hypoxic environment of tumor greatly limits the corresponding outcome [21–24]. To face this grand challenge, various solutions have been developed to fabricate new oxygenation nanomaterials [25–27]. Among of them, the introduction of oxygen-generating units triggered by Fenton/Fenton-like reaction was recognized to be one of the most promising approaches [28–30]. Therefore, the search for platforms involving Fenton/Fenton-like reaction based on porphyrin derivatives, which can relieve tumor hypoxia via oxygen-generating strategy, to achieve enhanced therapy outcome is an extraordinary desired target.

**Scheme 1:**
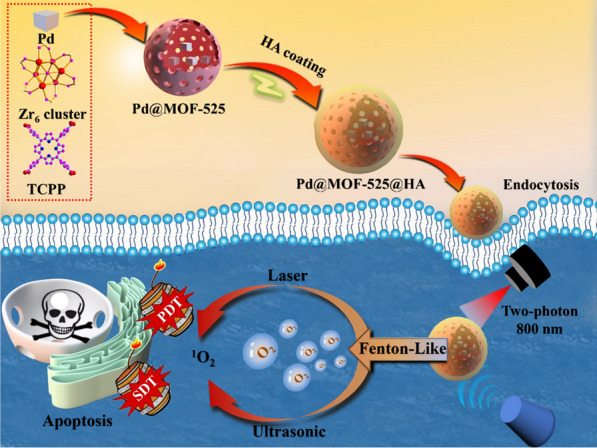
Scheme illustration showing the preparation of Pd@MOF-525@HA, highlighting the process of enhanced photodynamic and sonodynamic therapy

Based on the above considerations, we fabricated a porphyrinic MOF-based nanoplatform involving Fenton-like reaction, Pd@MOF-525@HA (Scheme 1), for enhanced oxygen-dependent anti-tumor therapy outcome. Thereinto, the porphyrinic MOF, namely MOF-525 (Zr_6_(OH)_4_O_4_(TCPP-H_2_)_3_) [31–33], was chosen as the target support based on the following considerations: (1) The porphyrin moiety within framework can act not only as PDT and SDT agents but also as two-photon responsive unit for near infrared (NIR) light-induced PDT with deeper tissue penetration due to its large π-conjugated system and rigid planar structure [34–36]; (2) The highly dispersed Pd nanocubes can be readily encapsulated, which can catalyze and react with over-expressed H_2_O_2_ in cancer cells to produce hydroxyl radicals (∙OH) and O_2_ through Fenton-like reaction, respectively, and then triggers cell apoptosis and greatly alleviates the tumor hypoxia for enhanced oxygen-dependent cancer therapy [37–40]; (3) The hyaluronic acid (HA)-wrapping through surface modification gives rise to considerable biocompatibility and cancer cell-specific targeting ability [41–43]. The results show that the obtained two-photon responsive nanoplatform, Pd@MOF-525@HA, possesses deeper tissue penetration, considerable light/ultrasonic-induced singlet oxygen (^1^O_2_) generation capacity, efficient oxygen generation and cancer cell specific targeting ability. Besides, we further combined two-photon fluorescence imaging to realize the combined precision anti-cancer treatment via PDT/SDT/CDT method.

## Results and discussion

**Fig. 1:**
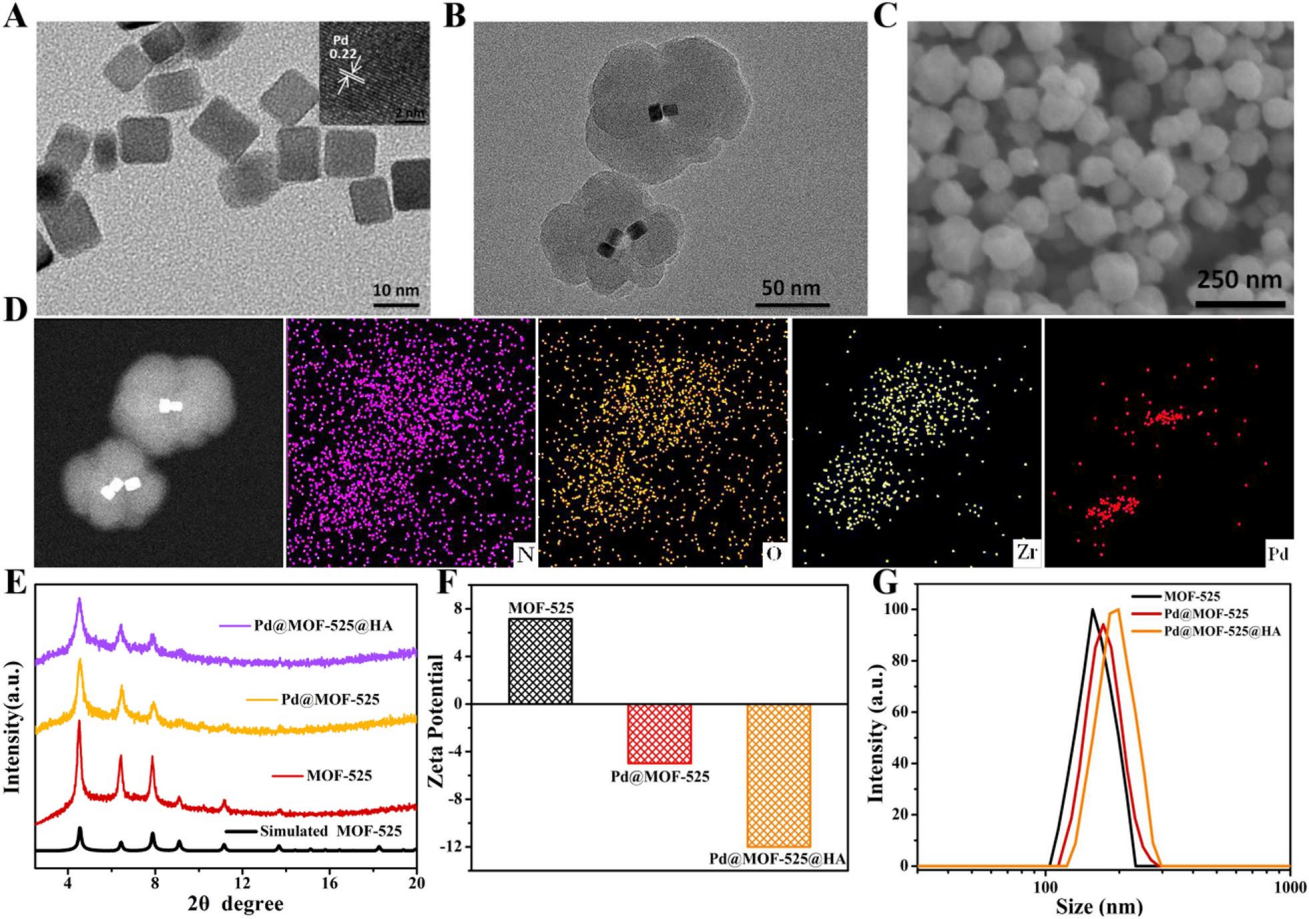
** A** TEM image of Pd nanocubes. Insert: HRTEM image of Pd nanocubes. **B** TEM image of Pd@MOF-525. **C** SEM image of Pd@MOF-525. **D** TEM elemental mappings of N, O, Zr and Pd of Pd@MOF-525. **E** PXRD patterns of simulated MOF-525, as synthesized MOF-525, Pd@MOF-525 and Pd@MOF-525@HA. **F** Zeta potential and **G** Hydrodynamic diameters of MOF-525, Pd@MOF-525 and Pd@MOF-525@HA

The composite Pd@MOF-525 with appropriate size for cellular uptake was constructed by introducing 5,10,15,20-tetracarboxy(4-carboxyphenyl)porphyrin (TCPP), pre-synthesized Zr_6_ clusters and Pd nanocubes via in-situ growth method [44]. As shown in Fig. 1A, the morphology of obtained Pd nanocubes was observed by transmission electron microscopy (TEM), which has a regular cubic structure, uniform size (≈ 10 nm) and good monodispersity. Moreover, the lattice spacing of 0.22 nm observed in the high-resolution TEM (HRTEM) image was assigned to the Pd (111) plane, confirming the structure of Pd nanocubes [45]. The TEM and scanning electron microscopy (SEM) images displayed clearly that Pd nanocubes were dispersed within MOF-525 and the size of Pd@MOF-525 increased to 130 nm (Fig. 1B, C). Simultaneously, the TEM elemental mappings were further performed for the Pd@MOF-525 composite, which proved that the homogeneous distribution of Pd element in the MOF-525 framework (Fig. 1D and Additional file 1: Fig. S1). Besides, the fabricated MOF-525 and Pd@MOF-525 were further demonstrated by the power X-ray diffraction (PXRD). These peaks could be matched with the parent framework of MOF-525 (Fig. 1E) and the characteristic peaks of Pd nanocubes appeared at high angles of 40.0 and 46.0 (Additional file 1: Fig. S2). In addition, HA was used to further modify the surface of Pd@MOF-525 to enhance its biocompatibility and cancer-specific targeting ability for further biological applications. The zeta potential analysis (Fig. 1F) manifested an obviously change in the surface charge from a positive potential of MOF-525 (+ 7.16 mV) to the negative potential of Pd@MOF-525 (− 4.98 mV) and Pd@MOF-525@HA (− 12 mV), corroborating the successful encapsulation of Pd nanocubes and wrapping of HA. Moreover, as shown by dynamic light scattering measurements (Fig. 1G), the average hydrodynamic diameters were reasonably enlarged from 156 nm (MOF-525) to 173 nm (Pd@MOF-525) and then to 197 nm for Pd@MOF-525@HA. All the above data results demonstrated the successful fabrication of Pd@MOF-525@HA. Notably, the size of Pd@MOF-525@HA maintained unchanged in serum-containing Dulbecco’s modified Eagle’s medium (DMEM, cell culture medium) within 7 days, which demonstrated the well stability of Pd@MOF-525@HA (Additional file 1: Fig. S5) in physiological conditions.

**Fig. 2:**
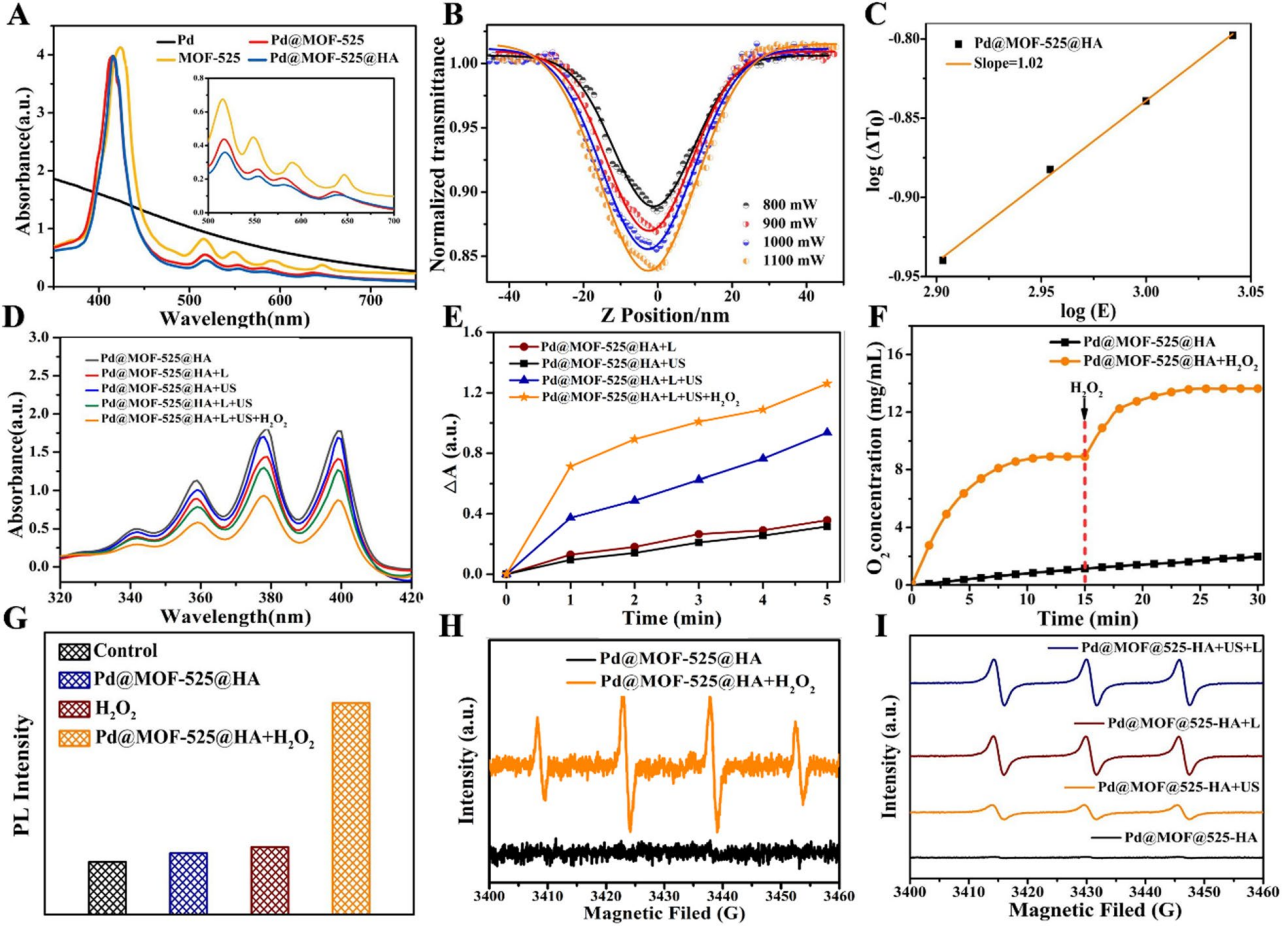
**A** UV-Vis absorption spectra of Pd, MOF-525, Pd@MOF-525 and Pd@MOF-525@HA. **B** Z-scan data at different pulse energy of Pd@MOF-525 (100 µg/mL) under the excitation at 800 nm. **C** The relationship between ΔT_0_ and E on a log-log scale excited by 800 nm laser. **D** ^1^O_2_ generation under different conditions detected by ABDA (Pd@MOF-525@HA:100 µg/mL, H_2_O_2_:100 µM). **E** Time-dependent generation of ^1^O_2_ (treated with different conditions) detected by ABDA. **F** The O_2_ concentration of solution treated with Pd@MOF-525@HA upon repeating addition of H_2_O_2_. **G** Detect the production of ∙OH with DCFH-DA under dark condition. **H** ESR signals of Pd@MOF-525@HA trapped by DMPO. **I** ESR signals of Pd@MOF-525@HA trapped by TEMP

The successful preparation of Pd@MOF-525@HA inspired us to further study its photophysical properties. From the absorption spectrum of  Pd@MOF-525@HA (Fig. 2A), the characteristic absorption for MOF-525 could be observed. First of all, considering the large π-conjugated feature of porphyrin moiety within frameworks, we investigated the reverse saturation absorption characteristics by z-scan experiments under near-infrared laser (800 nm) with different pulse energies (Fig. 2B) [46]. The results showed that the absorption intensity gradually enhanced with the pulse energy. Moreover, the relationship between changes of normalized (ΔT_0_, the minimum value of T_NL_(Z) curves) transmittance with laser pulse energy (E) was fitted in the log-log scale. As illustrated in Fig. 2C, the slope was calculated as 1.02 (the slope of the curve plus 1 is the number of effective photons absorbed) for Pd@MOF-525@HA, illustrating its two-photon absorption feature. It implies that Pd@MOF-525@HA can serve as an excellent candidate for NIR light-induced two-photon bioimaging.

Secondly, the light-induced ROS production ability of Pd@MOF-525@HA was studied systematically. As shown in Fig. 2D, the typical absorption of 9,10-anthracenediyl-bis(methylene)dimalonic acid (ABDA, ^1^O_2_ indicator) increased under white light (1 W cm^− 2^) or ultrasonic (1.2 W cm^− 2^) irradiation within 5 min, demonstrating the excellent ^1^O_2_ generation ability of Pd@MOF-525@HA. Notably, the well-dispersed Pd nanocubes within MOF-525 can convert H_2_O_2_ into O_2_ and then further improve the ^1^O_2_ generation ability of Pd@MOF-525@HA (Fig. 2E). Meanwhile, the oxygen production capacity of Pd@MOF-525@HA was measured by the dissolved oxygen meter to determine the concentration of O_2_ in the solution (Fig. 2F). It was found that the O_2_ concentration continued to increase after 15 min of repeated addition of H_2_O_2_, indicating that Pd@MOF-525@HA can continuously catalyze H_2_O_2_ to produce O_2_. On the other hand, the ROS fluorescent probe 2’,7’-dichlorofluorescein diacetate (DCFH-DA) was used to detect ∙OH under dark condition (Fig. 2G). The emission spectrum of DCFH-DA increased significantly after adding H_2_O_2_, which proved its excellent catalytic capacity of ∙OH production due to the catalytic decomposition of H_2_O_2_ [47]. Moreover, electron spin resonance trapping measurements using 5,5-dimethyl-1-pyrro-Line-N-oxide (DMPO) and 2,2,6,6-tetramethylpiperidine (TEMP) as the capture agent were further carried out to verify the type of produced ROS. The typical signal of DMPO-OOH (1:2:2:1 triplet) was observed after adding H_2_O_2_ into Pd@MOF-525@HA, indicating the production of ∙OH (Fig. 2H). In addition, the characteristic signals of 4-oxo-TEMPO (1:1:1 triplet) for Pd@MOF-525@HA was acquired under laser and ultrasonic irradiation, manifesting the production of ^1^O_2_ (Fig. 2I). In this sense, Pd@MOF-525@HA provides the possibility to relieve tumor hypoxia via oxygen-generating strategy for enhanced oxygen-dependent anti-tumor therapy.

**Fig. 3:**
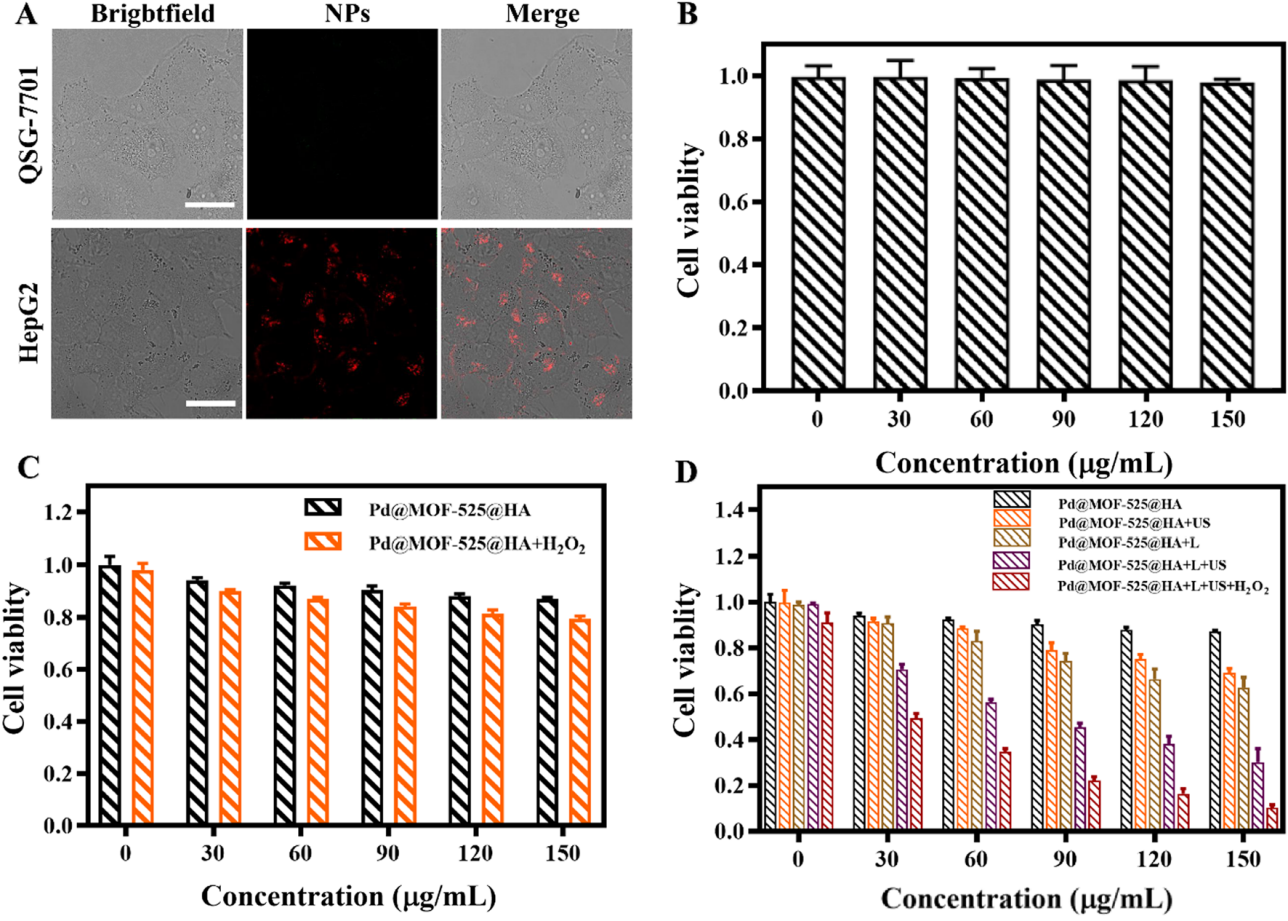
**A** CLSM images to verify the cellular uptake of Pd@MOF-525@HA (100 µg/mL) incubated with QSG-7701 cells and HepG2 cells (scale bar: 25 μm). **B** Cell viability of QSG-7701 cells incubated with Pd@MOF-525@HA. **C**, **D** Cell viability of HepG2 cells incubated with Pd@MOF-525@HA under different treatments (Laser: 800 nm, 1 W cm^− 2^; US: 1.2 W cm^− 2^; H_2_O_2_: 100 µM)

Encouraged by the two-photon feature and HA-wrapping Pd@MOF-525@HA, cellular uptake experiments with CD44 negative cells of human liver cells (QSG-7701) and CD44 positive cells of human liver cancer cell (tumor cells HepG2) were performed to evaluate its tumor cell-specific targeting and NIR light-induced fluorescence imaging ability. As shown in Fig. 3A, in comparison to the weak fluorescence of QSG-7701 cells incubated with Pd@MOF-525@HA under 800 nm laser irradiation, HepG2 cells group exhibited strong fluorescence. Moreover, the weak fluorescence of HepG2 cells pre-incubated with HA attributed to the preoccupation of receptor sites of CD44 cells by free HA (Additional file 1: Fig. S6) clearly demonstrated that HA-modified Pd@MOF-525 could effectively target tumor cells with CD44 receptors resulting in two-photon fluorescence imaging. Whereafter, the cytotoxicity of Pd@MOF-525@HA under different conditions was further researched by standard (4,5-dimethylthiazol-2-yl)-2,5-diphenyltetrazolium bromide (MTT) assay. As shown in Fig. 3B, C, the cell viability of QSG-7701 cells treated by Pd@MOF-525@HA was higher than that of HepG2 cells groups with/without H_2_O_2_ addition demonstrating that Pd@MOF-525@HA was harmless to normal cells and the generated ∙OH could give rise to decreased cell survival rate. As revealed by Fig. 3D and Additional file 1: Figs. S7–9, obviously cell apoptosis could be observed upon the light and ultrasound irradiation, and the cell viability was further decreased upon H_2_O_2_ added due to the production of O_2_. Notably, in the presence of H_2_O_2_, the survival rate of the cells treated by light and ultrasound irradiation was only 10%, which demonstrated the excellent in vitro PDT/SDT synergistic therapeutic outcome.

**Fig. 4:**
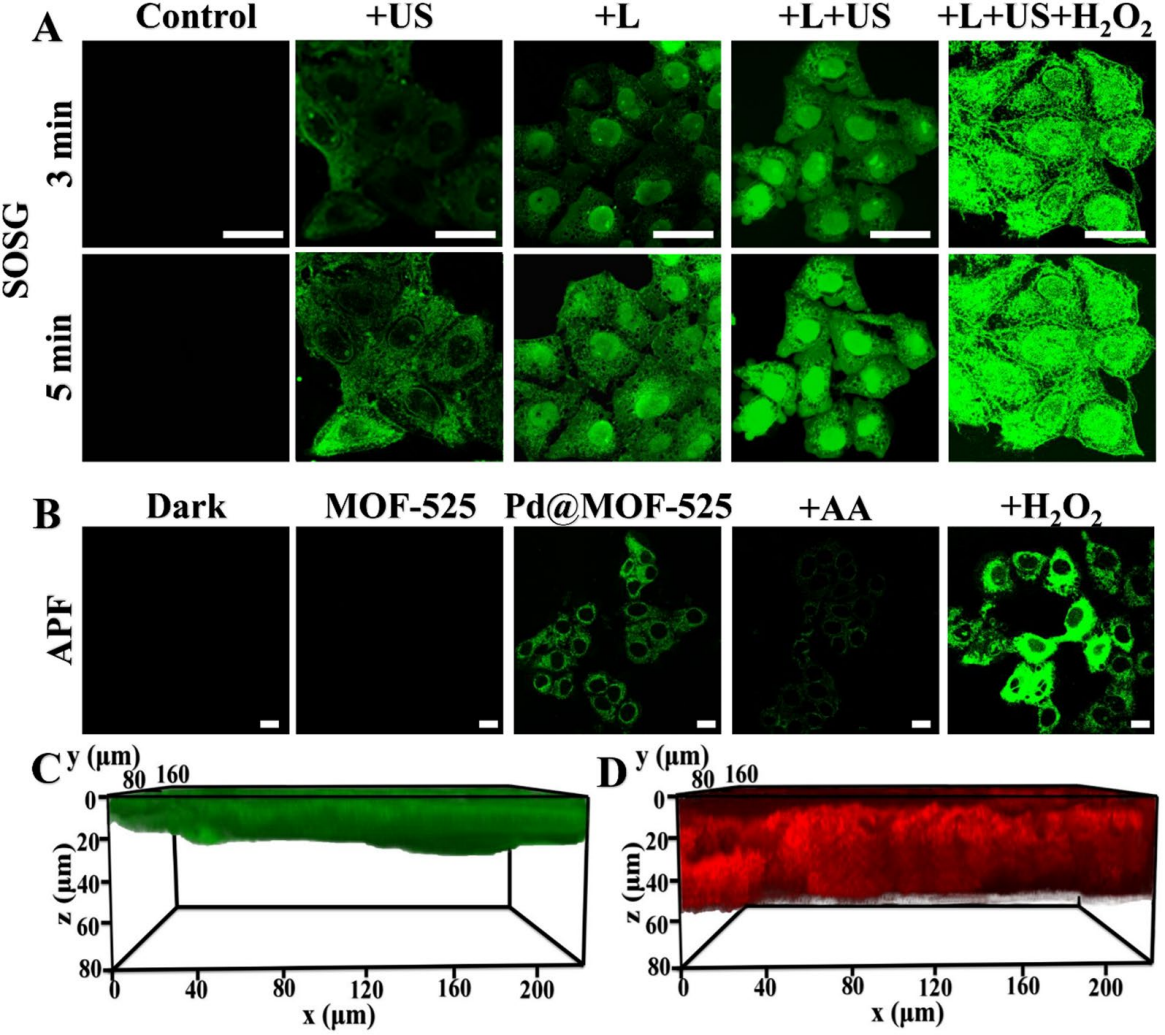
**A** CLSM images of HepG2 cells treated with Pd@MOF-525@HA (100 µg/mL) and detect the generation of ^1^O_2_ using SOSG (Laser: 800 nm, 1 W cm^− 2^; US: 1.2 W cm^− 2^; H_2_O_2_: 100 µM; Scale bar: 25 μm). **B** CLSM images of HepG2 cells treated with Pd@MOF-525@HA (100 µg/mL) and detect the generation of ∙OH using APF (AA: ascorbic acid, an inhibitor of ∙OH, 25 µM; H_2_O_2_: 100 µM; scale bar: 25 μm). **C** One-photon (458 nm, 0.2 W cm^− 2^) and **D** Two-photon (800 nm, 0.2 W cm^− 2^) 3D fluorescence images of tissue section

The in vitro PDT/SDT therapeutic effect was further evaluated through confocal laser scanning microscopy (CLSM) imaging. Firstly, the intracellular ^1^O_2_ and ∙OH generated from Pd@MOF-525@HA were detected by singlet oxygen sensor green (SOSG) and aminophenyl fluorescein (APF), respectively. As shown in Fig. 4A, obviously SOSG green fluorescence signal was collected with light/ultrasonic irradiation. Moreover, the enhanced SOSG signal from light and ultrasound worked together could be further improved when H_2_O_2_ added, which further unveiled that the light/ultrasonic induced ^1^O_2_ generation ability of Pd@MOF-525@HA could be effectively boosted by O_2_ production from Fenton-like reaction. Simultaneously, the obvious green fluorescence of APF was observed due to the generation of ∙OH (Fig. 4B) and the enhanced APF signal from H_2_O_2_ addition could be effectively quenched after adding ∙OH scavenger (ascorbic acid, AA). The above results corroborated the ROS generation ability of Pd@MOF-525@HA in cells for further treatment application. In addition, as demonstrated by the one- and two-photon fluorescence imaging in fixed mouse brain tissue (Fig. 4C, D), the deeper penetration depth (60 μm) of Pd@MOF-525@HA were collected upon 800 nm laser irradiation (two-photon fluorescence bioimaging). Notably, one-/two-photon fluorescence imaging of Pd@MOF-525@HA in mice displayed the same result. Compared to the images collected upon 458 nm irradiation (one-photon fluorescence bioimaging), brighter fluorescence signals could be observed under 800 nm laser irradiation. It demonstrated that the Pd@MOF-525@HA could be applied for two-photon fluorescence bioimaging and offer deeper penetration depth (Additional file 1: Fig. S10).

**Fig. 5:**
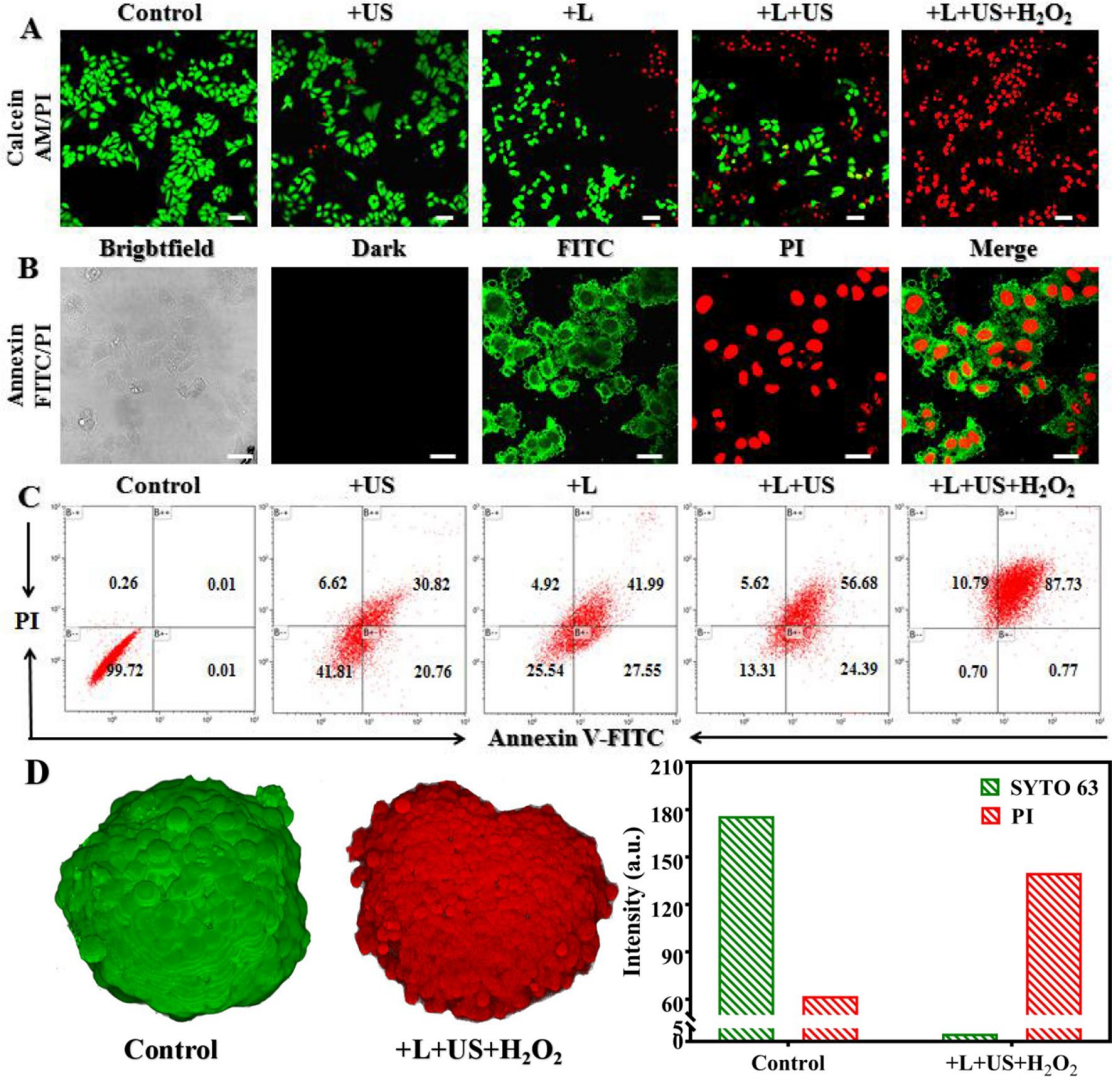
**A** CLSM images of HepG2 cells treated with Pd@MOF-525@HA after different treatment followed by stained with Calcein AM/PI (Laser: 800 nm, 1 W cm^− 2^; US: 1.2 W cm^− 2^; H_2_O_2_: 100 µM; Scale bar: 100 μm). **B** CLSM images of HepG2 cells incubated with Pd@MOF-525@HA for 12 h (Annexin V-FITC/PI were indicators of apoptosis, scale bar: 25 μm). **C** Cell apoptosis assays of HepG2 cells treated with Pd@MOF-525@HA after different treatment followed by stained with annexin V-FITC and PI. **D** 3D fluorescence images of MCTs after different treatments (Laser: 800 nm, 1 W cm^− 2^; US: 1.2 W cm^− 2^; H_2_O_2_: 100 µM)

Based on the deep tissue penetration and excellent intracellular ROS generation of Pd@MOF-525@HA, we deployed a thoughtful protocol to evaluate the efficacy of in vitro PDT/SDT therapy. The apoptosis was detected by using Calcein acetoxymethyl ester (Calcein AM, green) and propidium iodide (PI, red). As shown in Fig. 5A, brighter PI signal was collected when light and ultrasound worked together than that of only light or ultrasound treatment groups. Moreover, much brighter PI signal could be observed upon H_2_O_2_ addition demonstrating the enhanced in vitro PDT and SDT effect by alleviating hypoxia environment. The above results were further confirmed by Annexin V-FITC/PI treatment assay (Fig. 5B). In addition, the cell apoptosis was analyzed by flow cytometry using annexin V-FITC and PI as indicators under different treatment conditions. Upon light (800 nm, 100 mW cm^− 2^) and ultrasound (100 mW cm^− 2^) irradiation for 15 min, the fraction of late apoptotic cells was 56.68%, which was higher than that of only light (41.99%) or ultrasound (30.82%) treatment group (Fig. 5C) suggesting the outstanding synergistic therapeutic effect of Pd@MOF-525@HA due to its markedly enhanced ROS generation ability upon light and ultrasound worked together. With the addition of H_2_O_2_, the late apoptotic cells reached 87.73% suggesting enhanced oxygen-dependent therapeutic effect due to the generation of O_2_. Furthermore, HepG2 3D multicellular tumor spheroids of 3D cancer model (3D MCTs) were incubated with Pd@MOF-525@HA and stained with Calcein AM/PI to validate the efficiency in deep tissue. As shown in Fig. 5D, in contrast to the faint red fluorescence of the control group, MCTs formed an obvious necrotic core with bright PI fluorescence upon the NIR light and ultrasound irradiation corroborating the outstanding synergistic therapeutic effect. It manifested that Pd@MOF-525@HA can be employed as a PDT and SDT combination therapy platform guided by two-photon fluorescence imaging.

Furthermore, the systematically biosafety of Pd@MOF-525@HA was further evaluated by in vivo experiments. Firstly, we evaluated the effect of Pd@MOF-525@HA on red blood cells. As shown in Additional file 1: Fig. S11, even if the concentration of Pd@MOF-525@HA reached 100 µg/mL, it still showed ignorable influence on the integrity of red blood cells membranes or hemolysis. Secondly, it was proved by blood routine indexes test that no obvious toxicological effects were observed in healthy mice after intravenous injection of Pd@MOF-525@HA within 14 days (Additional file 1: Fig. S12). Moreover, the negligible changes of mice body weight further proved the good biosafety of Pd@MOF-525@HA (Additional file 1: Fig. S13). The above results suggested that Pd@MOF-525@HA could be an effective therapeutic agent for cancer therapy.

## Conclusions

In summary, an intelligent nanoplatform (Pd@MOF-525@HA) involving Fenton-like reaction was fabricated for enhanced oxygen-dependent anti-tumor therapy by oxygen-generating strategy. Thanks to the successful synthesis of the porphyrinic metal-organic framework, it can act not only as photo/sonosensitizers but also as two-photon responsive unit, the excellent ^1^O_2_ generation ability leads to considerable PDT and SDT outcome with deeper tissue penetration. In addition, the therapy effect can be further enhanced due to the O_2_ production from Pd nanocubes through Fenton-like reaction. It provides a promising platform to relieve tumor hypoxia via oxygen-generating strategy for enhanced oxygen-dependent anti-tumor therapy.

## Materials and methods

### Materials

All starting materials were obtained from commercial supplies and used without further purification. The chemicals of Sodium tetrachloropalladate(II) (Na_2_PdCl_4_), Sodium bromide (NaBr), 2′,7′-dichlorofluorescein diacetate (DCFH-DA), Ascorbic acid (AA) and Hyaluronic acid (HA) were purchased from Macklin Co., Ltd. The chemicals of acetone, polyvinylpyrrolidone (PVP, 58000w), *N*,*N*-Dimethylformamide (DMF), Ethanol and Acetic acid from Aladdin Co., Ltd. (4,5-dimethylthiazol-2-yl)-2,5-diphenyltetrazolium bromide (MTT) was obtained from Beyotime Biotech Co., Ltd. (China). Calcein AM/PI Kit and Annexin V-FITC/PI Apoptosis Detection Kit was obtained from Shanghai Bestbio (China). Ultrapure water was used throughout.

### Synthesis of pd nanocubes

PVP (53 mg), AA (30 mg), NaBr (130 mg) were dissolved into ultrapure water (4 mL), the mixture was then stirred at 80 ℃ over 5 min. 30 mg of Na_2_PdCl_4_ (dissolved in 1.5 mL of ultrapure water) was poured in the above mixture and stirred for 3 h. After being cooled to room temperature, Pd nanocubes were collected by centrifugation and then stored in 1 mL of DMF solution.

### Synthesis of Pd@MOF-525

Three mL of the synthesized Pd nanocubes were added to 2 mL DMF solution containing 100 mg Zr_6_ clusters), then the solution was stirred at room temperature for 4 h (solution A). TCPP(50 mg) was then dissolved into DMF solution (5 mL) and dispersed by ultrasound (solution B). Then acetic acid (6 mL) was added to mixed mixture of solution A and solution B, and stirred for 12 h. Finally, the product was collected after centrifugation and washing.

### Synthesis of Pd@MOF-525@HA

HA (10 mg) was dispersed in the ultrapure water (100 mL), 5 mg of Pd@MOF-525 was added after ultrasonic. After stirring for 24 h, washed with ultrapure water, and the final product was stored in ultrapure water.

### Apparatus

UV-Vis absorption spectra were recorded on a UV-265 spectrophotometer. SEM was detected by REGULUS8230*. TEM was carried on a JEM-2100. PXRD patterns were recorded on SmartLab 9KW. Fluorescence measurements were performed on a Hitachi F-7000 fluorescence spectrophotometer. O_2_ concentration was detected by dissolved oxygen meter (DO-958-S). One-photon and two-photon imaging data acquisition and processing were performed using Lecia TCS SP8 DIVE FALCON equipped with single-wavelength laser and femtosecond laser (adjustable output wavelength: 680–1080 nm, 80 MHz, 140 fs).

### Singlet oxygen (^1^O_2_) detection

The ^1^O_2_ was detected by 9,10-anthracenedipropanoic acid (ABDA, a singlet oxygen sensor) because the generated ^1^O_2_ would react with ABDA and reduce the absorbance around 378 nm. Pd@MOF-525@HA (50 µg mL^− 1^), ABDA (100 µM) and H_2_O_2_ (100 µM) were incubated together under white light and ultrasound (1.2 W cm^− 2^) irradiation within 0–5 min. The absorbance of the mixture was measured at the different time.

### Electron spin resonance (ESR) assay

The spin traps 2,2,6,6-tetramethylpiperidine (TEMP, trapping ^1^O_2_, 20 µL) and 5,5-dimethyl-1-pyrroline-N-oxide (DMPO, trapping ∙OH, 20 µL) were employed to detect the species of ROS generated by Pd@MOF-525@HA (50 µg mL^− 1^). The ESR signals of the Pd@MOF-525@HA before and after LED light (range from 400 to 700 nm, 40 mW cm^− 2^) and ultrasound (1.2 W cm^− 2^) irradiation were recorded.

### Cellular uptake analysis

QSG-7701 cells (CD44-negative) and HepG2 cells (CD44-positive) were seeded onto the cell culture dishes and grown to about 70% confluency for next use. QSG-7701 cells and HepG2 cells were treated with Pd@MOF-525@HA (100 µg mL^− 1^, and another dish HepG2 cells were precultured with 10 times of HA before incubation with Pd@MOF-525@HA. And after 8 h of incubation, the cellular uptake ability of Pd@MOF-525@HA was analyzed using CLSM.

### Cytotoxicity assays in cells

The PDT/CDT/SDT effect of Pd@MOF-525@HA was studied by the methylthiazolyldiphenyltetrazolium bromide (MTT) assay. The Pd@MOF-525@HA stock solution is diluted with fresh medium to the required concentration (0, 30, 60, 90, 120, 150 µg mL^− 1^). Before the experiment, HepG2 cells were cultured for 24 h in 96-well plates. Then exchange the cell culture medium with different concentrations of Pd@MOF-525@HA medium solution. They were incubated at 37 °C for 8 h in 5% CO_2_ atmosphere, and then irradiated by laser (800 nm, 1 W cm^− 2^) and ultrasound (1.2 W cm^− 2^) for 15 min. 100 µL of fresh medium were used to exchange the cell medium solutions 20 µL (5 mg mL^− 1^) MTT solution were added to each well following. The cell plates were then incubated for another 4 h. After removing the MTT medium, the formazan crystals were dissolved in DMSO (100 µL well^− 1^) and the absorbance was detected at 490 nm using a microplate reader. And duplicated experiments have been tested.

### Singlet oxygen detection in cells

HepG2 cells were treated with Pd@MOF-525@HA (100 µg mL^− 1^) for 8 h, and then incubated with 1 µM singlet oxygen sensor green (SOSG) for 10 min. Next, HepG2 cells were washed with PBS and irradiated for 15 min under laser (800 nm, 1 W cm^− 2^) and ultrasound (1.2 W cm^− 2^). The green fluorescence was observed by CLSM with the excitation wavelength of 504 nm (λ_em_: 500-550 nm).

### Live/dead assay with calcein AM/PI

After the HepG2 cells were washed with PBS solution twice, Pd@MOF-525@HA (100 µg mL^− 1^) was added to the above medium and incubated for 8 h, and then the cells were treated under different conditions. Calcein AM and PI were added to detect the cells vitality of HepG2 cells. Fluorescence images are collected by CLSM.

### Determination of annexin V-FITC and PI

HepG2 cells were incubated with Pd@MOF-525@HA (100 µg mL^− 1^) at 35 °C and 5% CO_2_ for 8 h. After adding H_2_O_2_, they were irradiated with laser (800 nm, 1 W cm^− 2^) and ultrasound (1.2 W cm^− 2^) for 15 min. Then, the Annexin V-FITC (1 µM) and PI (1 µM) were added and incubated for 20 min. Fluorescence images of the cells were collected by a confocal laser scanning microscope.

### Flow cytometry study

Cells seeded into the 6-well plates were incubated for 24 h, the medium containing Pd@MOF-525@HA (100 µg mL^− 1^) was used. After irradiated with laser (800 nm, 1 W cm^− 2^) and ultrasound (1.2 W cm^− 2^) 15 min, the cells were collected after centrifugation and then resuspended in binding buffer containing Propidium Iodide (PI, 10 µL) and Annexin-V FITC (5 µL) for 15 min in darkness. The signal was collected by a BD FACS Calibur flow cytometer (Beckaman/Gallios).

### The one/two-photon fluorescence imaging study of Pd@MOF-525@HA

A Lecia TCS SP8 DIVE FALCON which equipped with single-wavelength laser and femtosecond laser (adjustable output wavelength: 680–1080 nm, 80 MHz, 140 fs) was employed to achieve one/two-photon fluorescence imaging. HepG2 cells were treated with Pd@MOF-525@HA for 8 h. And then, slices were prepared from cardiac muscle tissue in Balb/c mice. The tissue sections were cut to 200 mm thickness. The tissue sections were incubated with Pd@MOF-525@HA for 30 min. The one-photon fluorescence emission was observed excitation at 458 nm (0.2 W/cm^2^). The two-photon fluorescence emission was observed excitation at 800 nm (0.2 W/cm^2^).

### Hemolysis assay

The mice red blood cells were collected from removing serum from the whole blood by centrifugation and washing. Whereafter, the Pd@MOF-525@HA was dispersed in phosphate-buffered saline with ascending concentration series (2, 5, 10, 20, 50, and 100 µg/mL), followed by adding into the mice red blood cells, respectively. Simultaneously, ultrapure water and phosphate-buffered saline were used as positive and negative groups, respectively. After the mixtures were incubated at 37 ℃ for 2 h and centrifugation at 2000 rpm for 10 min, the supernatant was collected and measured the absorbance at 540 nm. The hemolysis ratio was calculated using the following formula:

$$\text{hemolysis rate}\, (\%)=\frac{(\text{sample absorption - negative control absorption})}{(\text{positive control absorption - negative control absorption})}\times 100\%$$

A nanoplatform involving Fenton-like reaction, Pd@MOF-525@HA, to relieve tumor hypoxia *via* oxygen-generating strategy for enhanced oxygen-dependent anti-tumor therapy has been fabricated.

## Supplementary Information

**Additional file 1: Figure S1.** ^1^ H NMR spectrum of TCPP-OME (CDCl_3_). **Figure S2.** ^1^ H NMR spectrum of TCPP (DMSO-*d*_*6*_). **Figure S3.** TEM elemental mappings of C of Pd@MOF-525. **Figure S4.** PXRD patterns of Pd@MOF-525@HA nanoparticles. **Figure S5.** Hydrodynamic size distribution of Pd@MOF-525@HA for 7 days in serum-containing DMEM. **Figure S6.** CLSM images of HepG2 cells incubated with Pd@MOF-525@HA (Pd@MOF-525@HA treated with free HA in advance, scale bar: 25 μm). **Figure S7.** Cell viability of HepG2 cells incubated with Pd@MOF-525@HA under laser irradiation: 800 nm, 1 W cm^− 2^; H_2_O_2_: 100 µM; Pd@MOF-525@HA: 100 µg/mL). **Figure S8.** Cell viability of HepG2 cells incubated with Pd@MOF-525@HA under ultrasonic irradiation: US: 1.2 W cm^− 2^; H_2_O_2_: 100 µM; Pd@MOF-525@HA: 100 µg/mL). **Figure S9.** Cell viability of HepG2 cells incubated with Pd@MOF-525@HA under light and ultrasonic irradiation (Laser: 800 nm, 1 W cm^− 2^; US: 1.2 W cm^− 2^; H_2_O_2_: 100 µM; Pd@MOF-525@HA: 100 µg/mL). **Figure S10.** One/Two-photon fluorescence images after tail intravenous injection of Pd@MOF-525@HA (100 µg/mL). **Figure S11.** The images and hemolysis rates of red blood cells treated with different concentrations of Pd@MOF-525@HA. **Figure S12.** Blood biochemical and hematological analysis of the mice injected with Pd@MOF-525@HA after 14 days. **Figure S13.** Curves of body weight of mice after different treatments.

## Data Availability

All data generated or analyzed during this study are included in this published article and its additional files.

## Abbreviations

^1^O_2_: Singlet oxygen
PDT: Photodynamic therapy
SDT: Sonodynamic therapy
NIR: Near infrared
ROS: Reactive oxygen species
∙OH: Hydroxyl radicals
