# Supplementary material for: Two-photon responsive porphyrinic metal-organic framework involving Fenton-like reaction for enhanced photodynamic and sonodynamic therapy
Source: J Nanobiotechnology. 2022 May 6;20:217. doi: 10.1186/s12951-022-01436-3 (PMC9074235; doi:10.1186/s12951-022-01436-3)
Supplement: Supplementary file 1 — Additional file 1: Figure S1. 1 H NMR spectrum of TCPP-OME (CDCl3). Figure S2. 1 H NMR spectrum of TCPP (DMSO-d6). Figure S3. TEM elemental mappings of C of Pd@MOF-525. Figure S4. PXRD patterns of Pd@MOF-525@HA nanoparticles. Figure S5. Hydrodynamic size distribution of Pd@MOF-525@HA for 7 days in serum-containing DMEM. Figure S6. CLSM images of HepG2 cells incubated with Pd@MOF-525@HA (Pd@MOF-525@HA treated with free HA in advance, scale bar: 25 μm). Figure S7. Cell viability of HepG2 cells incubated with Pd@MOF-525@HA under laser irradiation: 800 nm, 1 W cm− 2; H2O2: 100 µM; Pd@MOF-525@HA: 100 µg/mL). Figure S8. Cell viability of HepG2 cells incubated with Pd@MOF-525@HA under ultrasonic irradiation: US: 1.2 W cm− 2; H2O2: 100 µM; Pd@MOF-525@HA: 100 µg/mL). Figure S9. Cell viability of HepG2 cells incubated with Pd@MOF-525@HA under light and ultrasonic irradiation (Laser: 800 nm, 1 W cm− 2; US: 1.2 W cm− 2; H2O2: 100 µM; Pd@MOF-525@HA: 100 µg/mL). Figure S10. One/Two-photon fluorescence images after tail intravenous injection of Pd@MOF-525@HA (100 µg/mL). Figure S11. The images and hemolysis rates of red blood cells treated with different concentrations of Pd@MOF-525@HA. Figure S12. Blood biochemical and hematological analysis of the mice injected with Pd@MOF-525@HA after 14 days. Figure S13. Curves of body weight of mice after different treatments. [file 12951_2022_1436_MOESM1_ESM.docx]

**Supporting Information**

**Two-photon responsive porphyrinic metal-organic framework involving Fenton-like reaction for enhanced photodynamic and sonodynamic therapy**

Wenyao Duan,^1†^ Bo Li,^1†^ Wen Zhang,^2†^ Jiaqi Li,^1^ Xin Yao,^2^ Yupeng Tian,^2^ Jun Zheng,^1*^ Dandan Li^1,2*^

^1^ Institutes of Physics Science and Information Technology, Key Laboratory of Structure and Functional Regulation of Hybrid Materials, Ministry of Education, Anhui University, Hefei 230601, P. R. China

^2^ Department of Chemistry, Key Laboratory of Functional Inorganic Material Chemistry of Anhui Province, Anhui University, Hefei 230039, P. R. China

^†^ Wenyao Duan, Bo Li and Wen Zhang contributed equally to this work

^*^ Corresponding author: J. Zheng (jzheng@ahu.edu.cn); D. Li ([chemlidd@163.com](mailto:chemlidd@163.com))


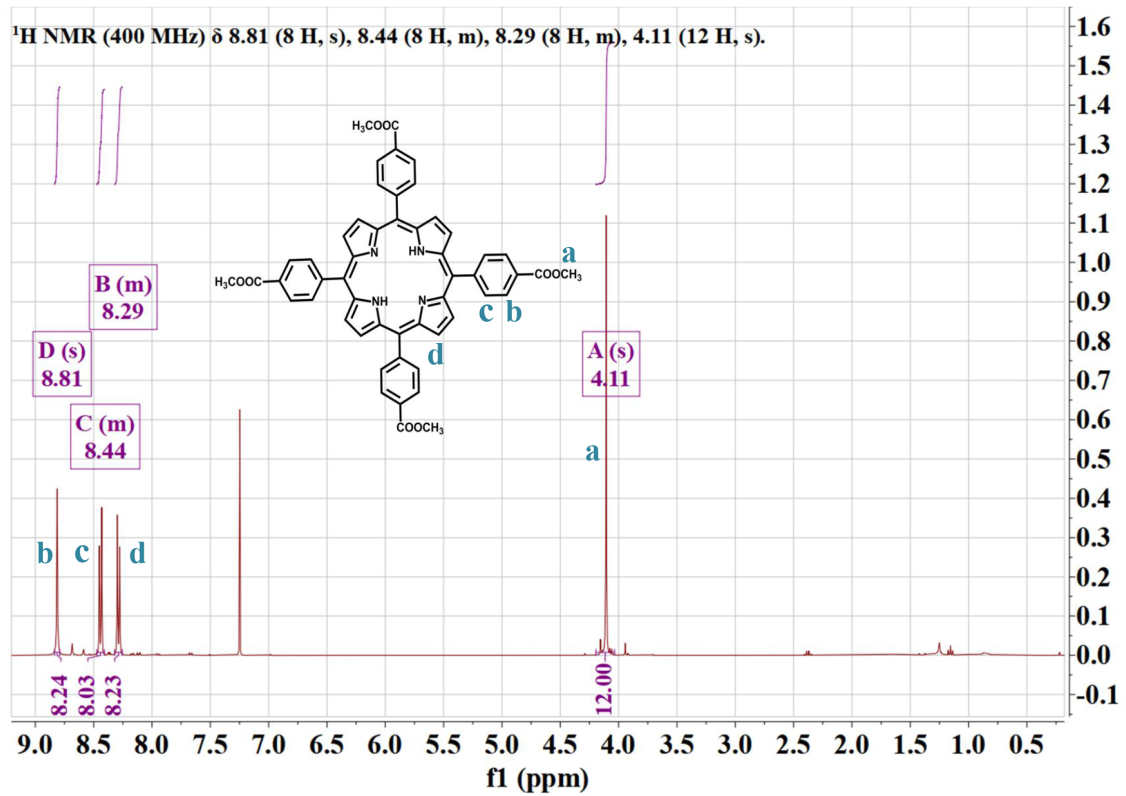


**Fig. S1** ^1^H NMR spectrum of **TCPP-OME** (CDCl_3_).


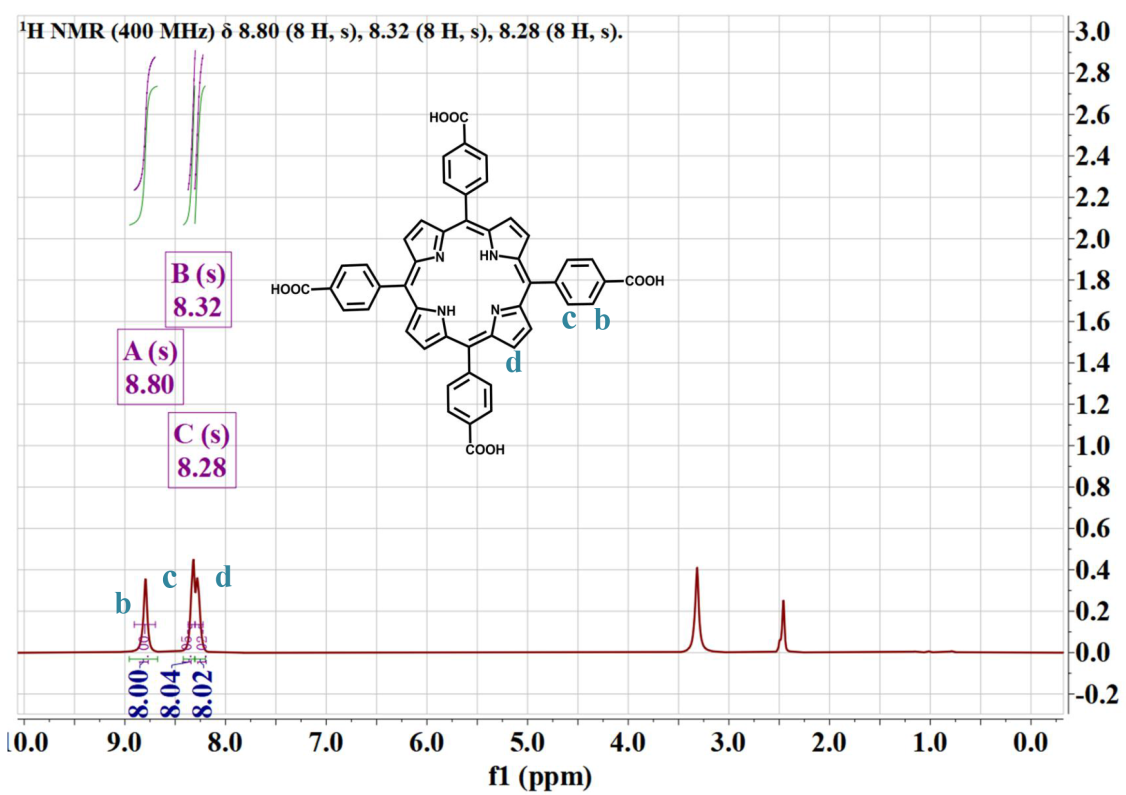


**Fig. S2** ^1^H NMR spectrum of **TCPP** (DMSO-*d_6_*).


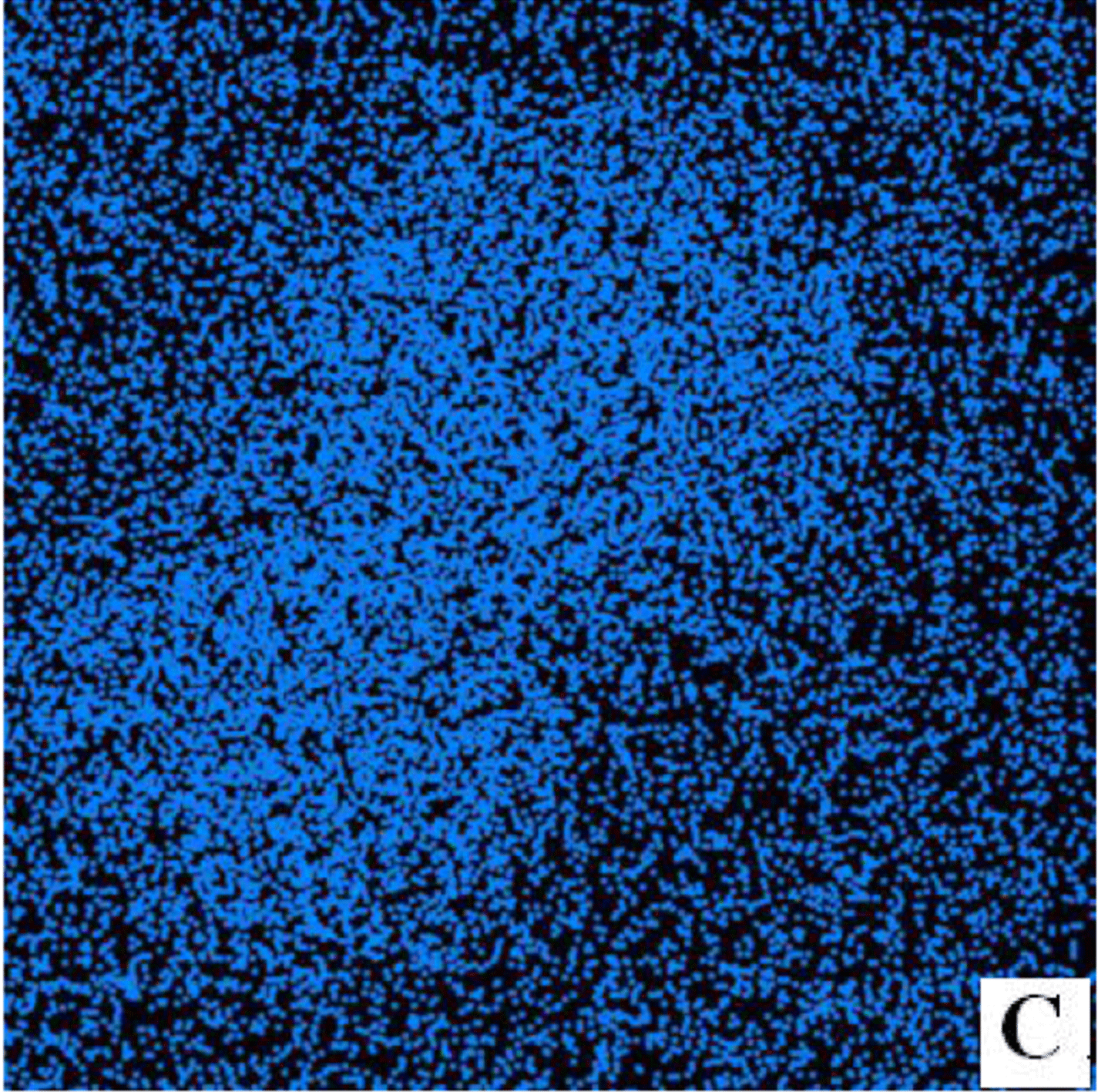


**Fig. S3** TEM elemental mappings of C of **Pd@MOF-525**.


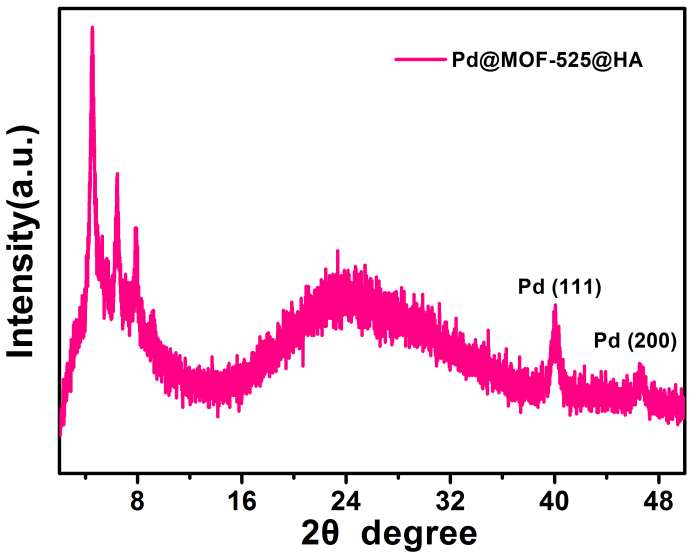


**Fig. S4** PXRD patterns of **Pd@MOF-525@HA** nanoparticles.

**
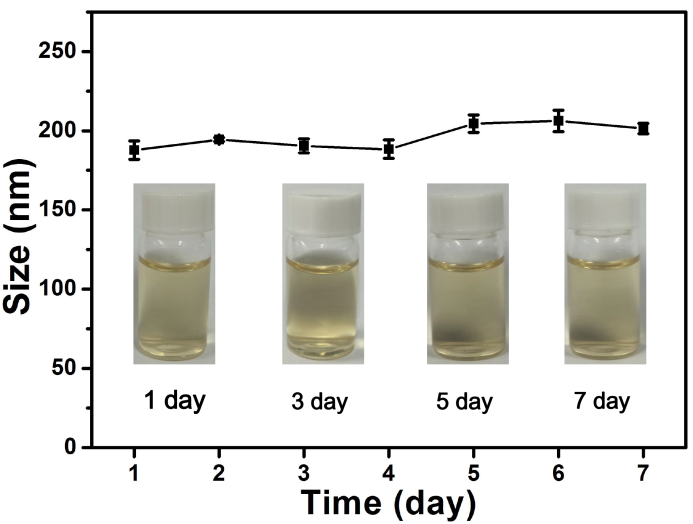
**

**Fig. S5** Hydrodynamic size distribution of **Pd@MOF-525@HA** for 7 days in serum-containing DMEM.


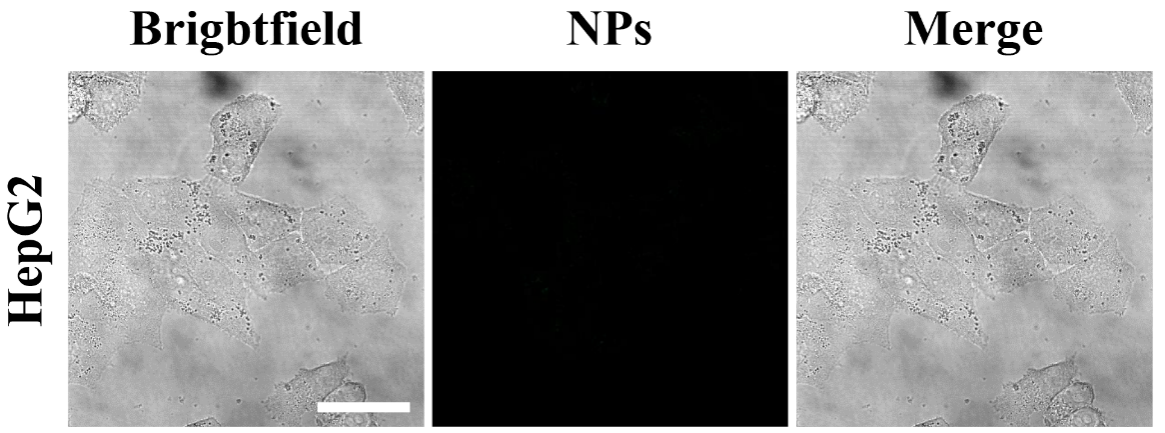


**Fig. S6** CLSM images of HepG2 cells incubated with **Pd@MOF-525@HA** (HepG2 cells treated with free HA in advance, scale bar: 25 μm).

**
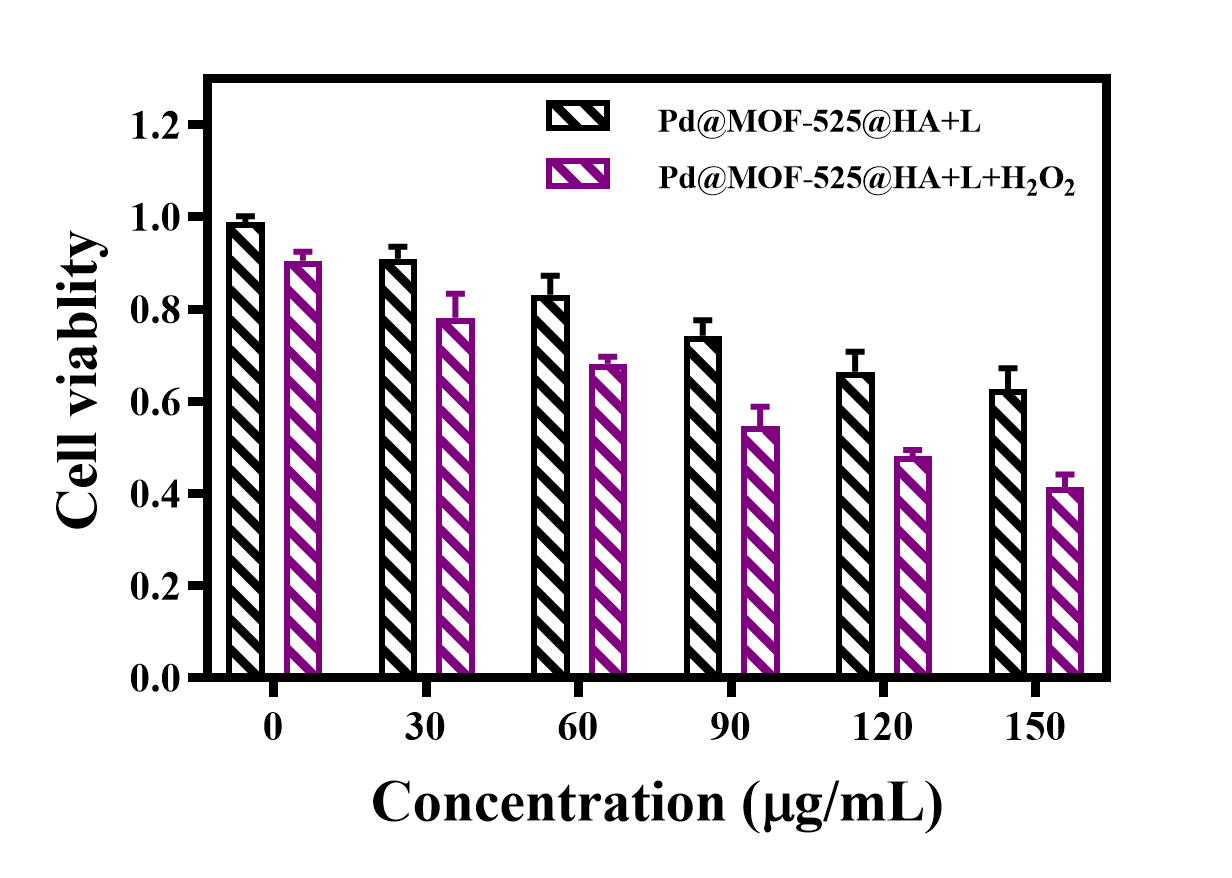
**

**Fig. S7** Cell viability of HepG2 cells incubated with **Pd@MOF-525@HA** under laser irradiation (800 nm, 1 W cm^-2^; H_2_O_2_: 100 μM; **Pd@MOF-525@HA**:100 μg/mL).

**
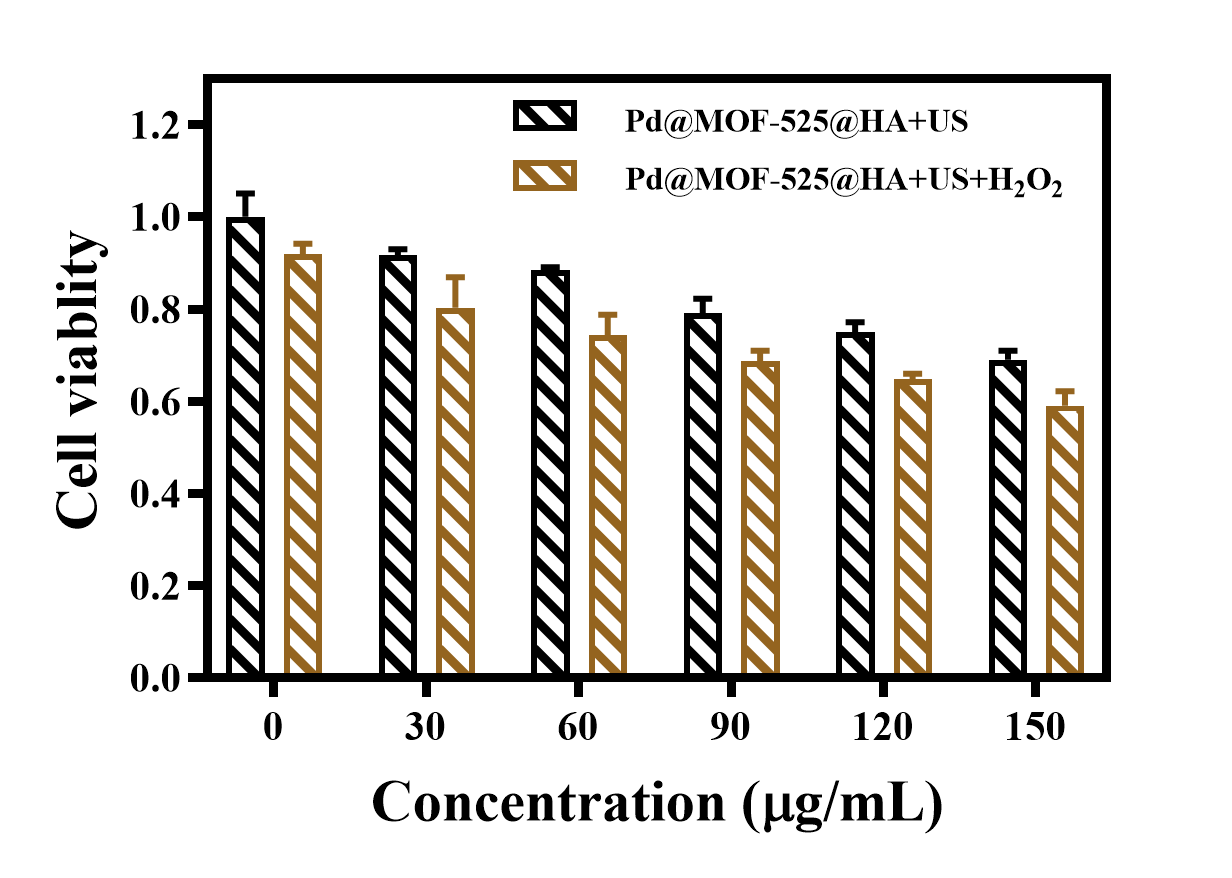
**

**Fig. S8** Cell viability of HepG2 cells incubated with **Pd@MOF-525@HA** under ultrasonic irradiation (US: 1.2 W cm^-2^; H_2_O_2_: 100 μM; **Pd@MOF-525@HA**:100 μg/mL).


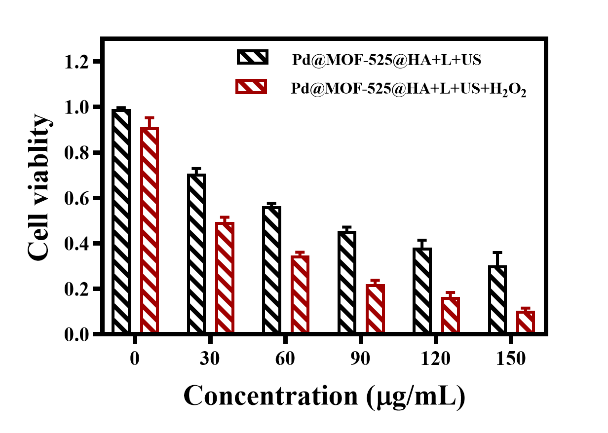


**Fig. S9** Cell viability of HepG2 cells incubated with **Pd@MOF-525@HA** under light and ultrasonic irradiation (Laser: 800 nm, 1 W cm^-2^; US: 1.2 W cm^-2^; H_2_O_2_: 100 μM; **Pd@MOF-525@HA**:100 μg mL^-1^).


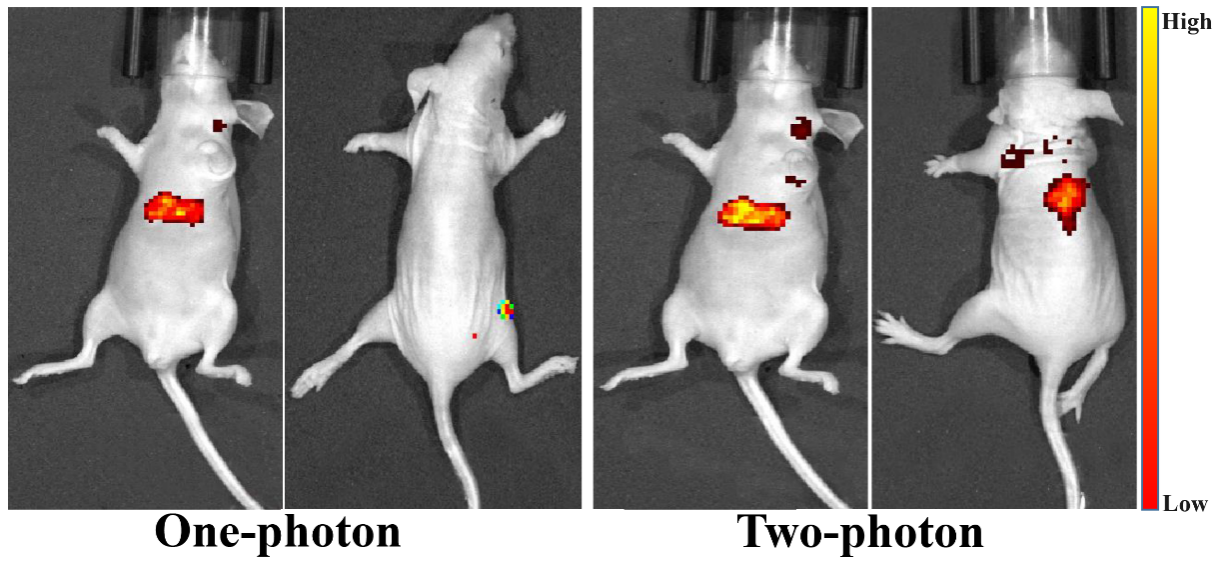


**Fig. S10** One/Two-photon fluorescence images after tail intravenous injection of **Pd@MOF-525@HA** (100 μg mL^-1^).


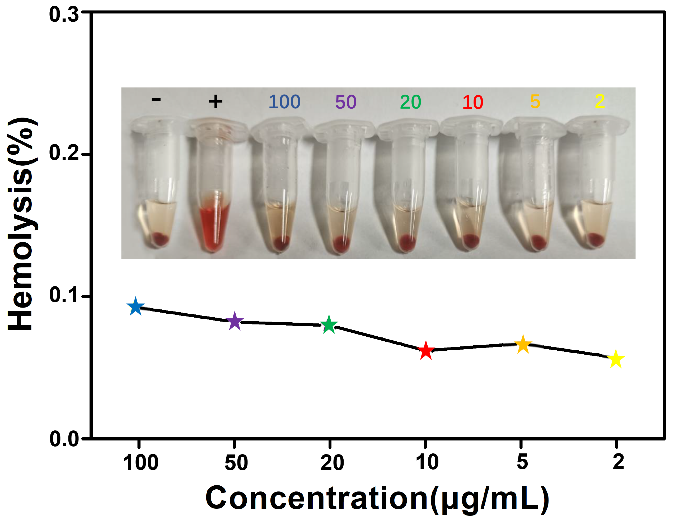


**Fig. S11** The images and hemolysis rates of red blood cells treated with different concentrations of **Pd@MOF-525@HA**.


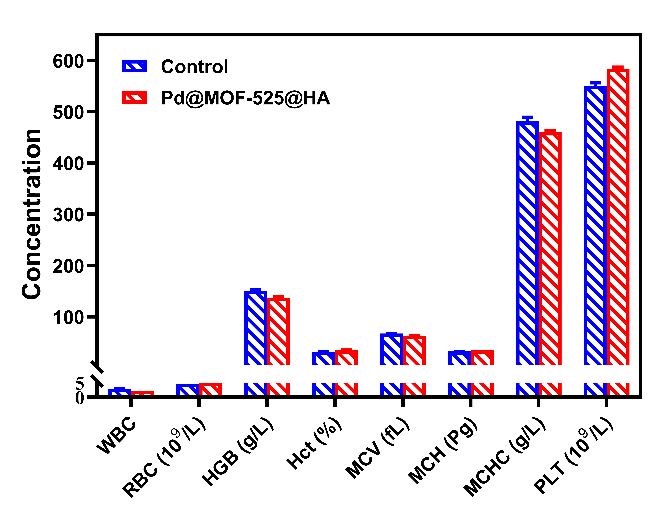


**Fig. S12** Blood biochemical and hematological analysis of the mice injected with **Pd@MOF-525@HA** after 14 days.


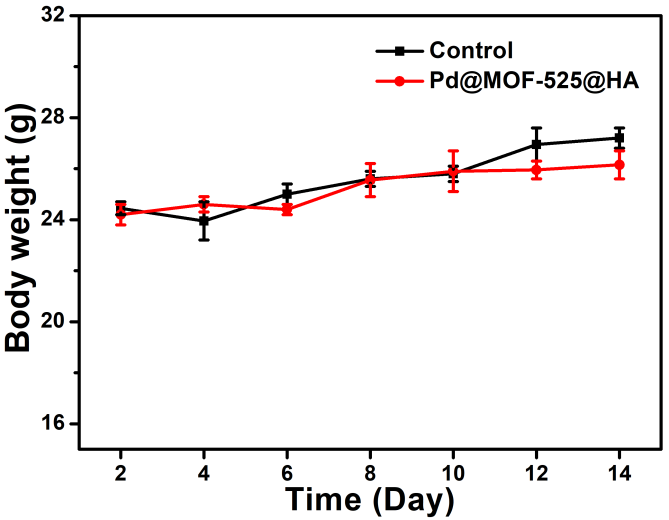


**Fig. S13** Curves of body weight of mice after different treatments.
